# Superior vena cava (SVC) reconstruction using autologous tissue in two cases of differentiated thyroid carcinoma presenting with SVC syndrome

**DOI:** 10.1186/1477-7819-7-75

**Published:** 2009-10-13

**Authors:** Nobuyuki Wada, Katsuhiko Masudo, Shohei Hirakawa, Tetsukan Woo, Hiromasa Arai, Nobuyasu Suganuma, Hideyuki Iwaki, Norio Yukawa, Keiichi Uchida, Kiyotaka Imoto, Yasushi Rino, Munetaka Masuda

**Affiliations:** 1 Department of Surgery, Yokohama City University Hospital, 3-9 Fukuura, Kanazawa-ku, Yokohama City, Kanagawa 236-0004, Japan; 2 Breast and Thyroid Surgery and Cardiovascular Center, Yokohama City University Medical Center, Minami-ku, Yokohama-shi, Kanagawa-ken 232-0024, Japan

## Abstract

Herein, we report two extremely rare cases of differentiated thyroid carcinoma (DTC) with extended tumor thrombus or mediastinum lymph node metastasis (LNM) involving the superior vena cava (SVC), causing SVC syndrome. Both of these patients were successfully treated with radical resection and reconstruction of the SVC using autologous tissue instead of an expanded polytetrafluoroethylene (ePTFE) graft. The left brachiocephalic vein was used to reconstruct the SVC in a papillary thyroid carcinoma patient with mediastinum LNM and a pericardial patch was used in a follicular thyroid carcinoma patient with tumor thrombus. Our search of the English-language literature found sporadic reports of SVC resection with reconstruction by vascular graft (ePTFE), interposed between the brachiocephalic vein and the right atrium. However, SVC reconstruction using autologous tissue in thyroid carcinoma has not been reported to date. To our knowledge, this is the first report describing such an unusual technique in DTC patients.

## Background

Superior vena cava (SVC) syndrome is extremely rare in patients with differentiated thyroid carcinoma (DTC). Direct primary tumor invasion (T4b tumor in the 6^th ^TNM classification), huge mediastinum lymph node metastasis (LNM), or extended tumor thrombus can be causes of such exceptionally unusual manifestations [1-9]. SVC syndrome leads to various clinical symptoms, such as headache, facial flush and swelling, and varicose veins over the upper body surface, finally resulting in lethal outcomes if appropriate treatment is not administered [1,4,9-11]. In particular, tumor thrombus in the great vein can also be a cause of sudden death due to pulmonary embolism. In the past, such advanced DTCs were usually treated with palliative management because of the difficulty of the surgical approach. Thus, surgery for SVC syndrome has been exceptionally challenging in only selected patients [7,12]. Recently, aggressive surgery has been performed occasionally to relieve SVC syndrome. Our search of the English-language literature found only sporadic reports of SVC resection and reconstruction with an expanded polytetrafluoroethylene (ePTFE) graft [2,3,8]. However, there are some concerns about the potential for vascular graft obstruction [8,13,14]. Our two patients were treated successfully with radical resection and reconstruction using autologous tissue, the left brachiocephalic vein or a pericardial patch, to reconstruct the interposition between the right brachiocephalic vein and the SVC. To our knowledge, this is the first report describing SVC reconstruction with autologous tissue to treat SVC syndrome in advanced DTC patients.

## Case presentation

### Case 1

**Figure 1:**
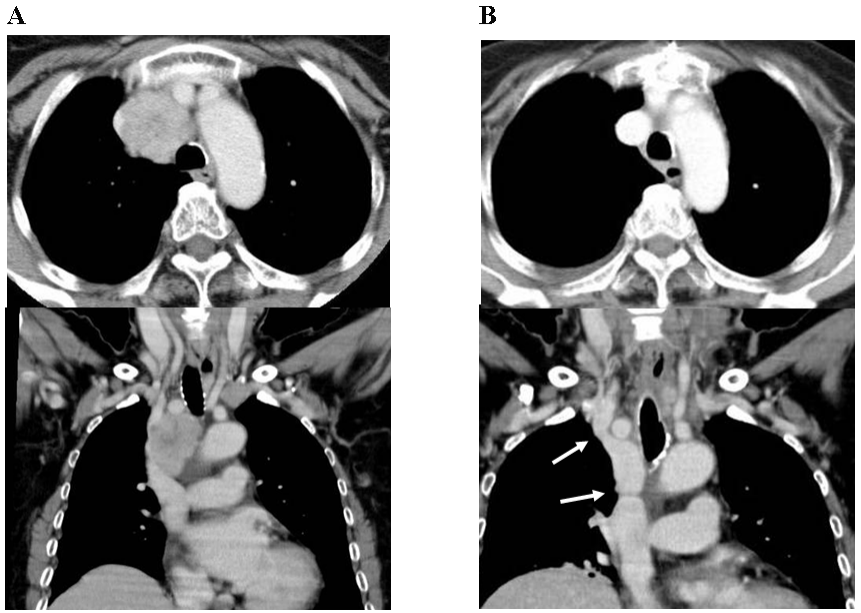
**A: Enhanced computed tomography (CT) reveals stenosis of the superior vena cava (SVC) due to invasion of the mediastinum lymph node metastasis (LNM)**. B: Postoperative CT scan shows the patency of the venous pathway after resection and reconstruction with the autograft (left brachiocephalic vein). Two arrows indicate the sites of distal and proximal anastomosis.

**Figure 2:**
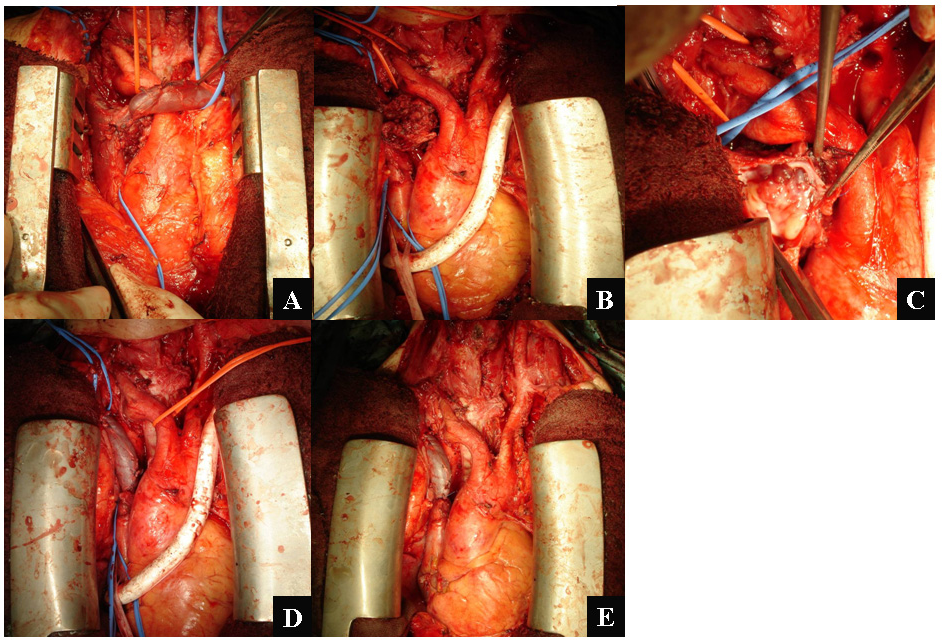
**A: Mediastinum LNM invading the posterolateral wall of right brachiocephalic vein and superior vena cava (SVC)**. B: Temporary bypass using an expanded polytetrafluoroethylene (ePTFE) graft was placed between left subclavian vein and right auricle after resection of the left brachiocephalic vein, which was not involved. C: Intraluminal invasion of mediastinum LNM in opened SVC. D: The right brachiocephalic vein and SVC were resected for complete removal of the invasive mediastinum LNM. The isolated left brachiocephalic vein was interposed to reconstruct the venous pathway between the right brachiocephalic vein and the SVC. E: Finally, the ePTFE graft as a temporary bypass was removed after confirmation of the flow in the reconstructed venous pathway.

A 74-year-old woman was referred to our institution for treatment of neck and mediastinum lymph node recurrence involving SVC. The patient initially underwent total thyroidectomy with bilateral modified neck dissection (MND) for primary papillary thyroid carcinoma (PTC) with cervical lymphadenopathy at another institution three years before our surgery. On our physical examination, only the cervical nodes were palpable. Computed tomography (CT) scan showed critical stenosis of the SVC due to the recurrent mediastinum LNM (Fig. 1A). This patient also presented with an elevated serum thyroglobulin (Tg) (707 ng/ml) without thyroglobulin antibody (TgAb) under TSH suppression (0.025 μIU/ml). Since the clinical symptoms from near total occlusion of SVC increased progressively, we subsequently performed radical resection to remove the recurrent mediastinum LNM and prevent further progression of SVC syndrome. Initially, we attempted to dissect only the mediastinum LNM with preservation of the great veins but could not remove such advanced lesions because of fixed invasion to the right brachiocephalic vein and SVC (Fig. 2A). Therefore, we subsequently attempted to perform radical resection followed by venous reconstruction. Temporary bypass using an ePTFE graft was placed between the distal site of the left brachiocephalic vein and the right auricle after resection of the left brachiocephalic vein (Fig. 2B). The left brachiocephalic vein was not involved macroscopically. After confirmation of the stable condition of the venous pathway under temporary bypass, the recurrent mediastinum LNM, right brachiocephalic vein and SVC were simultaneously resected. Intraluminal venous invasion of the mediastinum LNM was seen in the opened SVC (Fig. 2C). Next, vascular reconstruction was performed with the use of the already resected left brachiocephalic vein as autologous tissue (Fig. 2D). This autograft was placed between the distal site of the right brachiocephalic vein and the proximal site of the SVC. Finally, the ePTFE graft was removed after establishment of revascularization by autograft (Fig. 2E). There were no significant complications after this surgery.

Postoperative CT scan revealed that the reconstructed venous system was functioning well (Fig. 1B). Histopathological examination confirmed that the resected specimens were metastases from PTC and no portions of undifferentiated carcinoma were found.

Unfortunately, lung metastasis with pleural effusion occurred 13 months later. We could not provide any additional effective treatments to alleviate the symptoms. Her general condition gradually became worse and then she consequently died of disease 19 months after our surgery. Our surgical intervention was considered to contribute to preventing the development of SVC syndrome or immediate death by tumor embolism.

### Case 2

**Figure 3:**
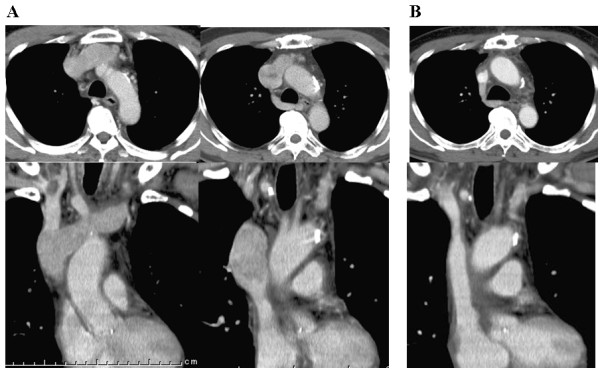
**A: Extended tumor thrombus totally occupying in the left brachiocephalic vein and the SVC was evident**. B: Successfully reconstructed venous pathway.

A 64-year-old man was referred to our institution for the treatment of SVC syndrome caused by extended tumor thrombus from a follicular thyroid carcinoma (FTC). The patient initially underwent total thyroidectomy with ipsilateral MND for primary FTC with cervical lymphadenopathy at another institution. This patient initially presented with tumor thrombus in the left internal jugular vein via the brachiocephalic vein to the upper part of the SVC. However, only the left internal jugular vein was simultaneously resected and the extended tumor thrombus in the left brachiocephalic vein and SVC was not removed during the initial surgery. Three months later, the patient complained of facial flushing and hypervascularity by varicose veins over the upper body surface, suggesting the occurrence of SVC syndrome. RI therapy was planned, however the clinical symptoms became worse during the preparation (levothyroxine withdrawal) for RI therapy. Therefore the preparation was discontinued and levothyroxine was again administered. The patient was subsequently referred to our institution an additional three months later (i.e., six months after the initial surgery) and additionally presented with right arm swelling and edema as clinical manifestations, suggesting progression of the SVC syndrome. No local or regional lesions were palpable on physical examination. Preoperative CT scan showed extended tumor thrombus totally occupying the left brachiocephalic vein and SVC (Fig. 3A). The patient had an elevated serum Tg (25000 ng/ml) without TgAb under TSH suppression (0.039 μIU/ml). Thus, the tumor thrombus that persisted after the initial surgery led to the progressive development of SVC syndrome.

**Figure 4:**
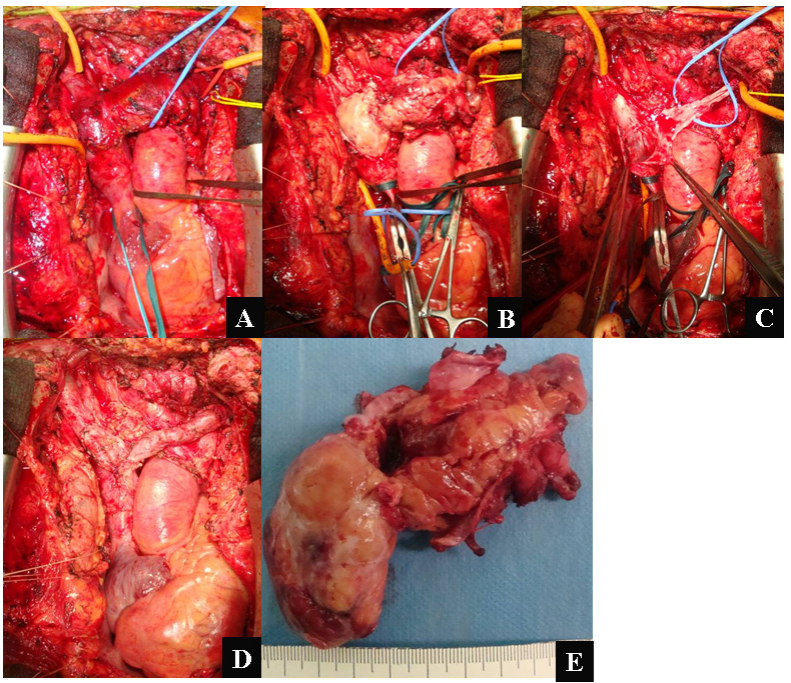
**A: Extended tumor thrombus in the left brachiocephalic vein and the SVC was apparent**. B: Tumor thrombus was macroscopically observed in the opened great veins. C: Thrombectomy alone was not possible because of the adhesion and invasion to the anterior intraluminal wall of the great veins; however a part of the posterior wall of these veins was able to be preserved. D: Pericardial patch was used to reconstruct the venous pathway between both brachiocephalic veins and the right atrium. E: Macroscopic finding of the resected tumor thrombus.

We immediately performed radical resection and reconstruction to entirely relieve the SVC syndrome. In our surgical procedure, the extended tumor thrombus was successfully removed through resection of the right and left brachiocephalic veins and the SVC (Fig. 4A, 4B). Indeed, isolated thrombectomy was not possible because of tumor adhesion and invasion to the anterior intraluminal wall of the great veins; however a part of the posterior wall of these veins could be preserved somewhat (Fig. 4C). An autologous pericardial patch was then used to reconstruct the venous pathway between both the brachiocephalic veins and the right atrium (Fig. 4D). Macroscopic findings of the resected tumor thrombus and veins are shown in Fig. 4E. Clinically, the SVC syndrome improved immediately after our surgery. Postoperative CT scan showed that revascularization was successfully achieved with the reconstructed venous system (Fig. 3B). Histopathological examination and subsequent immunohistochemical analysis revealed positive staining for Tg in the cells from the tumor thrombus, confirming that the resected specimens were angio-invasion from the FTC. After the surgery, RI ablation could be safely performed and TSH suppression is currently being maintained with an appropriate dose of levothyroxine. There has been no disease progression during the eight months of follow-up since our surgical treatments.

### Review of the literature

Table 1 summarizes the previous reports that describe the treatments and outcomes in DTC patients who exhibited extended tumor thrombus in the mediastinum great veins. The prognoses in such cases were principally unsatisfactory. However some treatments were effective in improving the progression of SVC syndrome and clinical outcomes. Thrombectomy was considered the most valuable surgical procedure when it was feasible. Our report is the first to use autologous graft as an alternative to the ePTFE graft that has generally been used for SVC reconstruction in DTC patients.

**Table 1:** Tumor thrombus from differentiated thyroid caricnomas in the mediastinum great veins.

**Authors**		**Patients**	**Pathology**	**Location**	**Therapy**	**Outcomes**	
Kim *et al*. [1]	1966	64M	FTC	IJV to RA	No surgery	Death	18 days

Thompson *et al*. [7]	1978	67F	FTC	IJV to RA	Thrombectomy	Alive	24 months

Perez *et al*. [12]	1984	48F	FTC	IJV to SVC	Thrombectomy	Alive	4 months

Niderle *et al*. [8]	1990	57M	FTC	IJV to RA	Thrombectomy	Alive	13 months

		79F	FTC	IJV to RA	Thrombectomy	Alive	50 months

		53F	FTC	IJV to RA	Reconstruction (ePTFE graft) Thrombectomy	Death	8 months

Patel *et al*. [4]	1997	79F	PTC	IJV to SVC, PV	Thrombectomy	Death	12 days

Onaran *et al*. [11]	1998	48M	Hürthle	IJV to SVC	No surgery (biopsy), RI therapy	Death	12 months

Wiseman *et al*. [10]	2000	84M	DTC?	IJV to SVC	Thrombectomy	Death	12 months

Koike *et al*. [16]	2002	26F	PTC	BCV to SVC	Cardiopulmonary bypass	Alive	8 months

Hasegawa *et al*. [15]	2002	78F	PTC	IJV to RA	Reconstruction (ePTFE graft)	Death	36 days

Motohashi *et al*. [3]	2005	64F	PTC	IJV to SVC	Reconsrtuction (ePTFE graft)	Alive	24 months

Sugimoto *et al*. [2]	2006	61M	PTC	BCV to RA	Thrombectomy, RI therapy	Death	12 days

Taib *et al*. [6]	2007	66F	FTC	IJV to RA	Thrombectomy, RI therapy	Alive	18 months

		62F	FTC	IJV to RA	Thrombectomy	Alive	18 months

		45F	FTC	IJV to BCV	Thrombectomy	Death	21 days

Yamagami *et al*. [17]	2008	74M	PTC	IJV to RA	No surgery, RI therapy, EBRT	Alive	7 months

Hyer *et al*. [5]	2008	81F	FTC	IJV to SVC	Reconsrtuction (pericardial patch)	Alive	66 months

Wada *et al*. [present]	-	64M	FTC	IJV to SVC	No surgery	Alive	7 months

PTC: papillary thyroid carcinoma, FTC: follicular thyroid carcinoma, ATC: anaplastic thyroid carcinoma, DTC: differentiated thyroid carcinoma, IJV: internal jugular vein, BCV: brachiocephalic vein, RA: right atrium, ePTFE: extended polytetrafluoroethylene (Gore-Tex) graft, RI: radioactive iodine, EBRT: external beam radiotherapy.

## Discussion

Since SVC syndrome caused by a mediastinum tumor from thyroid carcinoma is particularly rare, surgical intervention has been considered problematic and its indication remains controversial. Tumor thrombus within the SVC can be a cause of critical syndrome followed by lethal outcomes [1,4,7,10,11]. In the management of thyroid carcinoma, Thompson *et al*. firstly reported a case with successful resection of extended tumor thrombus in mediastinum great veins [7]. Perez *et al*. also reported a second case of successful resection of intraluminal SVC invasion [12]. Thus, the surgical approach to improve SVC syndrome has been challenged and SVC reconstruction has usually been performed with an ePTFE graft, interposed between the internal jugular vein and the right atrium, to reconstruct the venous pathway [2,3,8]. We performed SVC resection and reconstruction using autologous tissue without the use of an artificial vascular graft.

In general, patients with SVC syndrome die of disease when surgical intervention is not applied. Patel *et al*. and Wiseman *et al*. reported poor prognoses in patients without surgery [4,10]. Onaran *et al*. concluded that appropriate initial surgery might result in a disease-free state despite the residual presence of the tumor thrombus [11]. Niederle *et al*. reported three patients with SVC syndrome caused by tumor thrombus [8]. One patient underwent SVC reconstruction with an ePTFE graft, which was unfortunately occluded three months later, and another two patients were clinically asymptomatic after surgical treatment. Thus, aggressive surgery may be useful to relieve SVC syndrome and to prevent sudden death due to tumor embolism. Meanwhile, Hasegawa *et al*. reported immediate occurrence of intrapulmonary spread of the tumor after surgery with cardiopulmonary bypass, resulting in perioperative mortality due to respiratory failure [15]. Thus, surgical intervention for SVC syndrome remains controversial because of the treatment dilemma between perioperative morbidity and mortality with aggressive surgery and the poor prognosis with palliative therapy.

ePTFE graft has been used to improve SVC stenosis or occlusion and provide long relief from SVC syndrome [2,3]. Sugimoto *et al*. and Motohashi *et al*. recommended reconstruction of the SVC using an artificial graft as the treatment of choice [2,3]. However, there are some concerns about poor long-term patency of this artificial graft. Occlusion of the vascular graft has occasionally been reported during the follow up period [8,13,14]. Shintani *et al*. reported poor long-term patency of an ePTFE graft to reconstruct the left brachiocephalic vein [14]. They preferably advocate isolated reconstruction of the right brachiocephalic vein. The use of Y grafts was not recommended because of the frequent occlusion of such grafts. Alimi *et al*. indicated that close observation of the artificial graft is essential to determine the potential for graft problems earlier, especially during the first year after reconstruction [13].

A tumor thrombus can sometimes be relatively easily removed by thrombectomy alone without simultaneous resection of the great veins. Koike *et al*. reported a young woman with PTC who was successfully treated with thrombectomy from the brachiocephalic vein [16]. Yamagami *et al*. also reported a very rare case presenting with huge tumor thrombus extending to the atrium that was effectively treated with tumor thrombectomy alone, without harvesting all of the associated great veins [17].

Some authors have suggested that positive ring sign, a thin rim of contrast medium surrounding the tumor thrombus on enhanced CT examination, indicates the feasibility of successful tumor thrombectomy [6]. This sign may be useful to make a decision regarding the surgical strategy. Unfortunately, our case with extended tumor thrombus did not show this sign and thrombectomy alone was considered impossible because of the fixed adhesion and invasion of the tumor to the intraluminal wall of the great veins.

In our cases, we used autologous tissue, the brachiocephalic veins or a pericardial patch, to reconstruct the venous pathway. In fact, similar technique with autologous tissue has been used in the reconstruction of the SVC for other mediastinal malignancies [18,19]. In the field of cardiovascular surgery, these autologous tissues are preferably used as conduits, because of their low thrombogenicity, although the long-term patency of these materials in such patients is still unknown. We believe their long-term patency to be superior if tumor recurrence does not occur.

Endovascular therapy (EVT) may be primarily preferred as an initial treatment for SVC syndrome, because of the less invasiveness. Rizvi *et al*. concluded that EVT showed significant efficacy to relief the symptoms from SVC syndrome although surgical therapy remains for patients who are not eligible for EVT [20]. Charokopos *et al*. performed secondary EVT due to the thrombosis in a patient who underwent the SVC reconstruction with an ePTFE graft [21]. Thus, EVT may be considered useful less invasive treatment to improve SVC syndrome.

Table 1 summarizes the clinical results in DTC patients who presented with tumor thrombus in the mediastinum great veins. Both histological types, FTCs and PTCs, exhibited tumor thrombi and almost half of the patients died of disease. The patient's age and gender did not appear to affect clinical outcomes. More recently published review articles summarize the results of SVC reconstruction in other benign and malignant diseases. Lanuti *et al*. concluded that SVC resection and reconstruction were acceptable in the selected patients with SVC syndrome [22]. Picquet *et al*. concluded that SVC reconstruction could be safely performed as an alternative treatment in patients who did not respond to more conservative therapies [23].

Radiotherapy is effective in the fraction of thyroid carcinomas. Therefore, RI therapy and external beam radiotherapy (EBRT) are recommended to improve SVC syndrome when feasible. Hyer *et al*. reviewed the results from previous studies and recommended the use of various treatment modalities, such as surgery, RI therapy, and EBRT [5]. Taib *et al*. reported two patients who were successfully treated with thrombectomy followed by RI therapy [6]. Wilford *et al*. reported that EBRT contributed remarkably the improvement of SVC syndrome [9]. However, the elevation of serum TSH levels during the preparation period for RI therapy may adversely affect the growth of the tumor and may worsen the SVC syndrome. One of our patients experienced such progression of SVC syndrome due to elevated TSH during the preparation period. The preparation was immediately discontinued and this patient was then referred to our institution and subsequently underwent radical resection and SVC reconstruction using an autologous pericardial patch. After our surgery, RI treatment could be safely performed.

## Conclusion

To our knowledge, the use of autologous tissue has never been reported for SVC reconstruction in advanced DTCs patients. This approach might become the treatment of choice in surgical intervention for SVC syndrome because of the tolerance against infection and the anti-thrombogenicity of these materials compared with artificial grafts. Herein, we report the successful treatment of two DTC patients presenting with SVC syndrome.

## Consent

Written informed consent was obtained from the patients for publication of these case reports and any accompanying images. A copy of the written consent is available for review by the Editor-in-Chief of this journal.

## Competing interests

The authors declare that they have no competing interests.

## Authors' contributions

NW obtained written informed consent from the patients and drafted the manuscript. All authors carried out at least a part of operation and participated in collection of clinical data. NW and MM participated in the development of manuscript. All authors have read and approved the final manuscript.
